# Tumor-associated M2 macrophages promote prostate cancer invasion through the M-CSF-PCLAF pathway

**DOI:** 10.1371/journal.pone.0351858

**Published:** 2026-06-22

**Authors:** Yitian Ou, Chunwei Ye, Haiyang Jiang, Yong Zhu, Chengxing Xia, Delin Yang

**Affiliations:** 1 Urology Department, Kunming Medical University Second Affiliated Hospital, Kunming, Yunnan, China; 2 Urology Department, Yunnan Cancer Hospital, Kunming, Yunnan, China; Purdue University, UNITED STATES OF AMERICA

## Abstract

**Background:**

Prostate cancer (PCa), particularly in its advanced and castration-resistant forms, remains a major threat to men’s health, with the tumor microenvironment (TME) playing a crucial role in its progression. Tumor-associated macrophages (TAMs), especially the M2 phenotype, are key components of the TME. Our previous work identified that M2 TAMs in PCa upregulate M-CSF secretion via the MS4A6A-MYC pathway. This study aims to identify the critical downstream effector within PCa cells that mediates the tumor-promoting effects of M-CSF.

**Methods:**

The impact of M-CSF on PCa cell (PC3 line) viability, invasion, and migration was assessed using CCK-8, Transwell, and wound healing assays. To identify M-CSF-regulated downstream proteins, a comprehensive proteomic analysis (DIA-PASEF) was performed on PC3 cells treated with or without M-CSF. Bioinformatic analyses screened for differentially expressed proteins. Key candidate PCLAF was further validated using Western blot, analysis of TCGA-PRAD data, and immunohistochemistry on a prostate tissue microarray (79 patients). The functional role of PCLAF was confirmed through in vitro experiments and an in vivo xenograft model in nude mice, comparing tumors from PC3 control cells, PC3 cells overexpressing KIAA0101/PCLAF (PC3-KIAA0101+), and PC3 cells with local M-CSF injections.

**Results:**

M-CSF stimulation significantly enhanced PC3 cell viability, invasion, and migration in a concentration-dependent manner. Proteomic analysis revealed 95 differentially expressed proteins following M-CSF treatment. Among the top candidates, PCLAF/KIAA0101 was the only protein consistently and significantly upregulated by M-CSF in validation experiments. Analysis of TCGA data confirmed PCLAF’s significant overexpression in PCa tumors and its association with poorer disease-free survival. Tissue microarray analysis demonstrated that PCLAF expression was significantly higher in PCa tissues compared to benign tissues and positively correlated with higher Gleason Grade Groups and ISUP risk groups. In the xenograft model, both PC3-KIAA0101+ and PC3 + M-CSF groups exhibited significantly increased tumor growth, volume, and weight compared to the control group. IHC, Western blot, and PCR analyses of the xenograft tumors confirmed that PCLAF expression levels followed the pattern: PC3-KIAA0101+ > PC3 + M-CSF > control, and were positively correlated with tumor growth.

**Conclusions:**

This study indicates that M-CSF, secreted by M2 TAMs, promotes prostate cancer progression by upregulating the expression of PCLAF/KIAA0101 in cancer cells. PCLAF is overexpressed in PCa, correlates with tumor malignancy and poor prognosis, and its upregulation is sufficient to enhance tumor growth in vivo. These findings indicate the M-CSF-PCLAF axis as a key mechanism through which TAMs drive PCa invasion and progression, identifying PCLAF as a potential therapeutic target.

## Introduction

Globally, prostate cancer (PCa) is a common malignant tumor in men and has become the most prevalent and fastest-growing urological tumor in recent years [1]. According to the latest 2024 statistics in China, the number of new PCa cases nationwide has exceeded 3.3 million, with a growth rate now surpassing that of Western countries. Among these cases, only 30% are localized, while the rest are locally advanced or have distant metastases, posing a serious threat to men’s health [2–4]. Through treatments such as surgery, radiotherapy combined with endocrine therapy, neoadjuvant therapy, and targeted therapy, the prognosis of PCa patients has significantly improved. However, almost all patients progress to castration-resistant prostate cancer (CRPC) within two years, which carries a very poor prognosis [5].Therefore, elucidating the development mechanisms of PCa—particularly the molecular biological pathways responsible for drug resistance, immune escape, and subsequent poor treatment outcomes—holds significant scientific importance for improving existing advanced PCa treatment strategies and developing novel immune and targeted therapies for PCa.

Tumor-associated macrophages (TAMs) play an important role in various solid tumors. Different TAMs subsets coexist within tumors and carry distinct prognostic significance. A growing body of research supports this view, shifting the focus of tumor immunology from the intrinsic characteristics of tumor cells themselves to the composition and clonal alterations of the tumor microenvironment (TME) [6]. TAMs are involved in almost the entire process of tumorigenesis and development, including tumor angiogenesis, invasion, metastasis, colonization at distant metastatic sites, and the induction of immunosuppression [7–11]. Currently, key research on TAMs primarily focuses on the ratio between different polarization states, their expression patterns, and the association with poor prognosis. In our previous study, we employed different bioinformatics screening methods to identify key M2 macrophage genes most closely associated with prostate cancer (PCa) prognosis. We validated that M2 TAMs in the PCa tumor microenvironment upregulate MYC via MS4A6A, thereby promoting macrophages to secrete more M-CSF (CSF-1). We also confirmed that increasing concentrations of M-CSF enhance the cell viability, invasion, and migration capabilities of prostate cancer cells. In this part of our research, utilizing proteomics, bioinformatics analysis, and in vitro and in vivo experiments, we aim to further identify the sites within PCa cells that are regulated by M-CSF and to elucidate the pathways through which M-CSF promotes the initiation and progression of prostate cancer.

Omics analysis technology involves the comprehensive analysis of a specific class of molecules to screen for key markers within that category. Common omics technologies currently include genomics, proteomics, transcriptomics, and metabolomics, which play crucial roles in identifying differential genes and their interrelationships. Omics technologies also significantly contribute to cancer research: genomics and transcriptomics can increase screening coverage and reduce false positives, proteomics can screen for differentially expressed proteins corresponding to numerous transcriptional differences, and metabolomics can identify tumor microbial metabolism and interactions with the surrounding environment [12]. Proteomics currently plays an important role in studies of various solid tumors, including breast cancer, liver cancer, and early-onset gastric cancer, by: refining mechanisms related to metabolic abnormalities, viral infections, and gene mutations in tumorigenesis; discovering new biomarkers and potential targets to improve the efficacy of immune-targeted therapies; and determining specific tumor subtypes to enhance personalized treatment and prognosis monitoring [13–15]. Therefore, using proteomics to identify sites within prostate cancer cells that undergo significant changes following M-CSF regulation is central to explaining how TAMs promote prostate cancer initiation and progression via M-CSF.

The Proliferating cell nuclear antigen clamp-associated factor (PCLAF), also known as the KIAA0101 protein, and previously referred to as OEATC-1, is a binding regulator of the PCNA gene. Although PCLAF has been primarily studied in the context of DNA replication and repair, emerging evidence indicates its oncogenic role in multiple malignancies. In hepatocellular carcinoma, PCLAF overexpression correlates with poor prognosis and promotes proliferation via PCNA interaction. High PCLAF expression is associated with aggressive phenotypes and chemoresistance [16,17]. However, its role in prostate cancer remains largely unexplored. Notably, the M-CSF receptor (CSF1R) signaling pathway has been shown to regulate genes involved in cell cycle and DNA damage response in other cancer types. Given that PCLAF is a PCNA-associated factor and PCNA is a hub for DNA replication and repair, we hypothesized that M-CSF may upregulate PCLAF in prostate cancer cells, thereby promoting tumor aggressiveness. This study aims to test this hypothesis using proteomic screening followed by functional validation.

In our previous research, we screened and validated that TAMs in prostate cancer can upregulate M-CSF secretion via the MS4A6A-MYC signaling pathway. In this part of the study, we used proteomics to screen for downstream proteins that exhibited significant expression differences after M-CSF treatment of prostate cancer cells. Through bioinformatics and expression validation experiments, we ultimately identified the PCLAF gene, which showed significant changes in expression and function. Furthermore, we validated, both in vitro and in vivo, that M-CSF-mediated upregulation of PCLAF promotes prostate cancer progression.

## Materials & methods

### Cell line co-culture

PC3(Authenticated by Shanghai Biowing Applied Biotechnology Co. LTD.) was selected as PCa cell line and THP-1 as monocyte cell line.The PC3 and THP-1 used in the experiment were all cultured in 10% fetal bovine serum(40130ES76, Yeasen Co. LTD.) with 1640 medium(11875168, Gibco™), and then cultured in a cell incubator of 37°C and 5% carbon dioxide after adding 100U/ml Penicillin-Streptomycin Solution(BL505A, Biosharp Co. LTD.).We used THP-1 cells to induce M0 macrophages by adding PMA(100ng/ml, 48h; Cat. No. HY-18739, MedChemExpress), and continued to add IL-4(20ng/ml, 48h; Cat. No. HY-P70445, MedChemExpress) and IL-13(20ng/ml, 48h; Cat. No. HY-P70779, MedChemExpress) to induce M2 macrophages.

### Cell phenotypic experiment

CCK-8 assay: Cell supernatant was removed from the culture plate, and 100ul basic medium was added to each well. After setting up the blank control, we added 100ul basic medium and 10ul cck-8 solution to each hole. After 2 hours of incubation in the dark, the OD value of each hole was detected at 450nm by an enzyme labeling instrument. Cell viability% =(experimental OD-blank hole OD)/(control hole OD- blank hole OD)*100%.

Wound healing assay: The cells were digested and collected by trypsin, and the cell concentration was adjusted to 2.5 × 10^4^/ml. The cells were inoculated into the Ibidi scratch plug-in. The left and right holes of the plug-in were inoculated with 100ul cell suspension. The plug-in were removed when the overnight culture reached the fusion degree of 95%. After washing, the photos were taken at 0h, 12h and 24h.

Transwell invasion: Matrigel matrix glue was melted at 4°C. The gun heads, Transwell chambers and 24-hole plates should be precooled at 4°C. The whole process of glue laying should be operated on ice. Then 50ul Matrigel was added to the Transwell film(8μm). The Matrigel should be evenly spread on the Transwell film and dried 30 min at 37°C. Cell concentration was adjusted to 1 × 10^5^ /ml with the culture supernatant, and the 100ul cell suspension was inoculated into the Transwell-matrigel chambers. The 3 holes in each group were repeated and incubated for 24 hours. Transwell chambers were put into 1 ml 4% polymethyl fixative. After removing the upper chamber liquid, we dripped 200ul 4% polymethyl fixative to the upper chamber for 30 minutes. The fixing solution was removed and the chambers were put into 500ul crystal violet dye solution for 15 minutes. After 3-times washing by PBS, the results were photoed under the microscope.

### Protein extraction and processing for pxroteomics

Protein samples were stored at −80°C. For detection, the samples were taken out, and four volumes of RIPA lysis buffer (containing 1% protease inhibitor and 8M urea) were added to each group, followed by ultrasonic lysis. The samples were centrifuged at 12,000 × g for 10 minutes at 4°C to remove cell debris. The supernatant was then transferred to a new EP tube, and the protein concentration was determined using a BCA kit.

Equal amounts of protein from each sample were taken for enzymatic digestion. The volume of each sample was adjusted to the same level using lysis buffer, and trichloroacetic acid (TCA) was slowly added to a final concentration of 20%. The mixture was vortexed and precipitated at 4°C for 2 hours. After centrifugation at 4,500 × g for 5 minutes, the supernatant was discarded, and the precipitate was washed 2–3 times with pre-cooled acetone. Once the precipitate was air-dried, tetraethylammonium bicarbonate (TEAB) was added to a final concentration of 200 mM, and the precipitate was resuspended using ultrasonication. Trypsin was added at a protease-to-protein ratio of 1:50 for overnight digestion. The following day, dithiothreitol (DTT) was added to a final concentration of 5 mM, and the reduction was carried out at 56°C for 30 minutes. Subsequently, iodoacetamide (IAA) was added to a final concentration of 11 mM, and the mixture was incubated at room temperature in the dark for 15 minutes.

The peptides were dissolved in mobile phase A for liquid chromatography and separated using a NanoElute ultra-high-performance liquid chromatography system. Mobile phase A consisted of 0.1% formic acid and 2% acetonitrile in water, while mobile phase B was composed of 0.1% formic acid in acetonitrile-water. The liquid chromatography gradient was set as follows: 0–14 minutes, 6%–24% B; 14–16 minutes, 24%–35% B; 16–18 minutes, 35%–80% B; 18–20 minutes, 80% B, with a flow rate of 500 nl/min. After separation by the ultra-high-performance liquid chromatography system, the peptides were ionized using a Capillary ion source, and data were acquired using a timsTOF Pro2 mass spectrometer. The ion source voltage was set to 1.75 kV, and both the parent peptides and their secondary fragments were detected and analyzed by the TOF system. The data acquisition mode was set to data-independent acquisition parallel accumulation-serial fragmentation (dia-PASEF). Each primary mass spectrum required 20 PASEF acquisitions, with a scanning range of 300–1500 m/z. The secondary mass spectrum range was set to 400–850 m/z, with a window of 7 m/z.

### Proteomics analysis

In proteomics analysis, experimental methods such as Hallmark gene annotation, Reactome annotation, and GO analysis overlap with the methodologies described in the first section on bioinformatics. Therefore, this section will provide detailed descriptions only of the non-overlapping parts.

After obtaining the raw files from mass spectrometry analysis, the screening of key regulatory proteins follows the procedure below:

(1) First, a sample-specific protein database is constructed based on the specific source of the samples, followed by database searching using analysis software.
(2) Quality control analysis is performed on the results obtained from the database search, both at the protein and peptide levels.
(3) Quantitative analysis of proteins is conducted, including quantitative distribution and reproducibility analysis, while also displaying the distribution of quantitative intensity values across samples.
(4) Functional annotation of the identified proteins is carried out, including annotations for: GO, KEGG, Protein domain, COG/KOG, STRING database, Reactome, WikiPathways, Hallmark, and transcription factor (TF) annotation. Among these, Reactome, WikiPathways, Hallmark, and transcription factor annotations are only available for specific species in relevant bioinformatics analyses.
(5) Based on the quantitative results, the fold change (FC) and T-test significance P-value are calculated for comparisons between two groups. Differential screening is performed according to set thresholds, and statistical graphs related to differential analysis are generated. If there are three or more sample groups, one-way analysis of variance (ANOVA) is also applied to calculate the significance P-value for multiple groups. Differential proteins across multiple groups are screened based on the ANOVA P-value for subsequent analysis.
(6) Functional classification and statistical analysis of differentially expressed proteins between two groups are performed, including GO secondary classification, subcellular localization classification, COG/KOG classification, and KEGG pathway classification statistics.
(7) Enrichment analysis of differentially expressed proteins between two groups is conducted using Fisher’s exact test, covering functions such as GO, KEGG, Protein domain, Reactome, and WikiPathways.
(8) When the project involves multiple experimental groups, enrichment clustering analysis is performed to compare the functional associations of differentially expressed proteins under different experimental conditions.
(9) Through protein-protein interaction (PPI) network analysis, key regulatory proteins under specific experimental conditions are identified.

### KEGG, protein domain, COG and Transcription factor annotation

Utilizing the Kyoto Encyclopedia of Genes and Genomes (KEGG) database allows for the integration of currently known protein-protein interaction information, which includes data on genes and gene products (Gene database), pathways and related complexes (Pathway database), as well as biological complexes and associated reactions (Compound and Reaction databases). We employed the KEGG database to annotate protein pathways in proteomics, using the BLAST method (with parameters set to blastp, e-value ≤ 1e-4) to compare the identified proteins. For each sequence’s BLAST results, the highest-scoring match was selected for annotation.

A protein domain refers to a conserved amino acid sequence within a protein, typically composed of 25–500 amino acids, that can function independently as a structural or functional unit. In the obtained project data, we performed protein domain annotation for the identified proteins based on the Pfam database and the PfamScan tool.

The Cluster of Orthologous Groups of proteins (COG) is divided into the prokaryotic COG database and the eukaryotic KOG database. We used the EggNOG database to conduct more comprehensive homology-based classification of a broader range of protein sequences, and systematically performed phylogenetic tree construction and functional annotation for each homologous gene cluster.

Using the annotated protein gene information obtained, we employed the TRRUST and GTRD public databases—which are based on transcription factors and target genes—to annotate the gene information of transcription factors corresponding to the detected proteins.

### Subcellular localization

Due to differences in the binding characteristics of proteins to membrane structures, proteins in eukaryotic tissue cells are localized to various organelles and cellular components. We performed subcellular localization annotation of the identified proteins using WolF Psort software.

### Ethics statement

(1) Human Subject Research: The Medical Ethics Committee of the Second Affiliated Hospital of Kunming Medical University has approved this research and the approval number is Shen-PJ-Ke-2025–348.
(2) Animal Ethics and Study Approval: The Ethics Committee of Yunnan Cancer Hospital has approved this research and the approval number is SLKYLX2025−05.

The above studies have received ethics committee approval for their research protocols and informed consent procedures. According to the submitted review documents, the studies plan to obtain written informed consent from participants.

### Personnel training

All experimental procedures were conducted by researcher Ou Yitian holding valid laboratory animal training certificates (Nos. 2021A055). Training encompassed animal handling, sterile technique, recognition of pain and distress, administration of substances, and humane euthanasia methods.

### Animal housing

Thirty (30) male Balb/c-nude mice (5 weeks old, average weight 25 g) were purchased from Vital River Laboratories (Beijing, China) and housed in a specific pathogen-free (SPF) facility (NO.422023600009814). Animals were acclimatized for one week prior to experiments under controlled conditions: temperature 22 ± 2°C, humidity 50 ± 10%, and a 12-hour light/dark cycle. Sterilized standard rodent chow and acidified water (pH 2.5–3.0) were provided ad libitum. Cage enrichment (nesting material, shelters) and social housing were provided to minimize stress.

### Xenograft tumor experiment in nude mice

Male nude mice(Balb/c-nude SPF class, Beijing Weitong Lihua Experimental Animal Technology Co., Ltd. Hubei Branch) were purchased and housed in an SPF-grade animal facility. The mice were housed at a density of 5 mice per IVC cage. The SPF animal room temperature was maintained at 22 ± 2°C with a relative humidity of 40%−60%. The experimental animals had free access to water and food, and the experiment commenced after a 3-day acclimatization period.

Preparation of Cell Suspension for Inoculation:

On the day of inoculation, the cell suspension for inoculation was prepared in advance on the cell culture bench. The specific steps were as follows:

(1) Discard the culture medium supernatant, add 5 mL of PBS to each culture dish for washing, then add 3 mL of trypsin for digestion for 5 minutes.
(2) After observing under a microscope that the cells were mostly detached, add 6 mL of 1640 medium to each dish to terminate the digestion, and gently pipette to detach any remaining adherent cells.
(3) Collect the cells into a 10 mL EP tube and centrifuge at 1000 rpm for 5 minutes at room temperature.
(4) Using the inoculation density and volume calculated in S1 Table as the target, resuspend the cells in serum-free 1640 medium.
(5) Perform cell inoculation in nude mice. The selected inoculation site was the right axilla. Disinfect the area three times from the inside out using alcohol swabs. Draw the cell suspension into an insulin syringe and perform a subcutaneous injection in the right axilla according to the total cell suspension volume calculated in S1 Table. Ensure all the cell suspension is injected before slowly withdrawing the needle. Carefully observe the injection site for any leakage before returning the mouse to its cage for continued housing.

### Xenograft experiment data collection

After tumor cell inoculation, monitor tumor growth daily at fixed times and record the impact of tumor growth on the normal behavior of the nude mice. Carefully record any abnormalities occurring during the experiment. Primary monitoring includes: survival status of nude mice, weight gain or loss, food and water intake, activity level, and condition of fur, eyes, or any other abnormalities. Starting 5–7 days post-inoculation, measure the body weight of the nude mice and changes in tumor size every 3 days. Tumor volume is calculated using the formula: Tumor Volume (mm^3^) = 1/2 (Longest diameter in mm × Shortest diameter^2^ in mm^2^). A tumor growth curve is plotted accordingly.

### In vivo fluorescence imaging

During tumor formation, monitor the tumor status in nude mice using in vivo fluorescence imaging. The specific steps are as follows:

(1) Turn on the small animal in vivo imaging system and the IVIS system. Wait until the CCD temperature drops to −90°C before starting the experiment. Set the imaging mode to bioluminescence mode.
(2) Inject D-luciferin sodium salt intraperitoneally into the nude mice (150 mg/kg). Perform in vivo imaging 15 minutes post-injection. Anesthetize the mice using isoflurane gas before imaging.

### Humane endpoints and euthanasia

Humane endpoints were strictly defined and implemented to prevent severe suffering. Animals were euthanized immediately (within 1–2 hours of confirmation) if they met any of the following criteria:

(1) Tumor volume ≥ 1500 mm^3^.
(2) Body weight loss ≥ 20% from baseline.
(3) Clinical signs of severe distress, including inability to access food or water, prolonged immobility, or other marked behavioral abnormalities.

No animals died prior to meeting these endpoints during the planned study. Euthanasia was performed by cervical dislocation under deep isoflurane anesthesia, consistent with the American Veterinary Medical Association (AVMA) Guidelines. At the experimental endpoint (planned tissue collection), all remaining surviving animals were euthanized using the same method.The whole animal experiment took 16 days(From 13th May,2024–26th May, 2024).

### Sample collection

The specific steps are as follows:

(1) After the nude mouse is anesthetized, remove it from the anesthesia induction chamber and arrange the dissection instruments.
(2) Euthanize the nude mouse by cervical dislocation, place it on the operating surface, and carefully disinfect the tumor dissection site in the axilla using alcohol swabs and gauze, repeating three times.
(3) Use ophthalmic scissors to cut the skin, then use forceps to bluntly dissect the tumor.
(4) Use scissors to open the thoracic and abdominal cavities. Use forceps to grip the diaphragm, open the left and right sides of the diaphragm, remove the tissue on the surface of the liver, and then completely remove the liver. Next, use forceps to gently lift the mouse lungs, excise the lungs and heart, and carefully dissect any adhering surface tissue.
(5) Use scissors to carefully separate the renal capsule and remove the kidneys. Use scissors to carefully separate the mesentery and surface fat, then excise the visceral fat.
(6) After sample collection is complete, completely dissect the tumor, weigh it, and store it at −80°C. Dispose of the mouse carcass using appropriate biohazard procedures.

### Prostate cancer grading and grouping methods

(1) Grade Group Classification:
  Grade Group 1: Gleason Score ≤ 6
  Grade Group 2: Gleason Score 3+4=7
  Grade Group 3: Gleason Score 4+3=7
  Grade Group 4: Gleason Score = 8
  Grade Group 5: Gleason Score > 8
(2) ISUP Risk Group Classification:
  ISUP Low Risk: Gleason Score = 6
  ISUP Intermediate Risk: Gleason Score = 7, 8
  ISUP High Risk: Gleason Score = 9, 10

### Immunohistochemistry

Paraffin sections were dewaxed with xylene. Hydration was carried out with concentrated water and different concentrations of ethanol. Then we used 0.01 mol/l PBS to rinse 3 times. Firstly, the slices were soaked in sodium citrate buffer. Then microwave was used to heat. After cool down to room temperature, the heating process was repeated twice. The non-specific binding sites were blocked with serum. 20μl primary antibody was added to each slice and incubated overnight at 4°C. Filter paper was used to absorb the excess primary antibody, then we added second antibody 20μl to each slice and incubated at 37°C for 2 hours, and washed the slices with PBS 3 times. DAB hydrogen peroxide was used for coloration and hematoxylin was used for re-dyeing. Then the slices were soaked in ethanol for 2 min. Xylene was used to make the slices transparent. Finally, we used Aipathwell(Wuhan Servicebio technology COm, LTD. China) to analyze.

Human prostate tissue array for IHC was bought from Zhongkeguanghua Co.,ltd. Item No. M079Pr01.

### Polymerase chain reaction(PCR)

RNA was extracted using TRIZOL and reagents were prepared before starting assay procedure. 1μl sample was taken to measure the concentration of RNA in ND-1000 spectrophotometer. The first strand of cDNA is synthesized by RNA reverse transcription. After repeated sampling of the target gene and the internal reference gene, LightCycle96 was put in to denaturation, annealing and extension. After the reaction, the Ct value was calculated and analyzed.

### Western Blot(WB)

Western Blot protocol consists of the following key steps:

(1) Protein Extraction and Denaturation: Cells are lysed using RIPA buffer containing inhibitors at 4°C. The supernatant is collected after centrifugation, mixed with Loading Buffer, and boiled for protein denaturation before storage at −20°C.
(2) Protein Quantification (BCA Assay): A protein standard curve is prepared. Samples and standards are incubated with BCA working solution at 37°C. The absorbance at 562nm is measured using a microplate reader, and the sample protein concentration is calculated based on the standard curve.
(3) SDS-PAGE Electrophoresis: Based on the calculated concentrations, samples are loaded with a total protein amount of 40 μg. A gel is prepared with a lower separating gel and an upper stacking gel. Electrophoresis is performed under constant voltage (first 60V then 90V) until the tracking dye reaches the bottom.
(4) Membrane Transfer: Proteins are transferred from the gel to a PVDF membrane. A “sandwich” assembly is used, and transfer is carried out at a constant current of 200mA for 1 hour in an ice bath.
(5) Immunoblotting (Antibody Incubation): The PVDF membrane is sequentially processed as follows: blocked with 5% skim milk; washed with TBST; incubated with a specific primary antibody at 4°C overnight; washed with TBST; incubated with an HRP-conjugated secondary antibody at room temperature for 2 hours; and given a final wash with TBST.
(6) Detection (Chemiluminescence): ECL chemiluminescent substrate (prepared by mixing solutions A and B in equal proportions) is applied to the PVDF membrane. The chemiluminescent signal is detected and captured using a gel imaging system.

## Results

### Effects of different M-CSF concentrations on PCa cells

**Fig 1:**
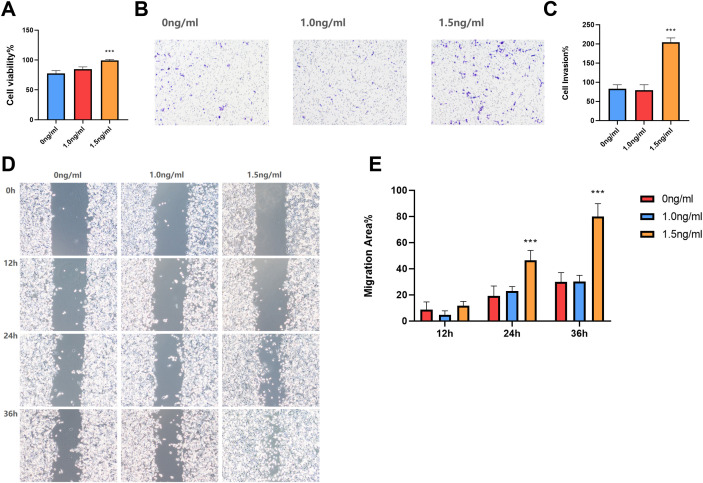
Effect of M-CSF on the cell viability, invasion ability and migration ability of PC3 cells. **(A)**With the increasing level of M-CSF, the viability of PC3 cells showed a corresponding upward trend(P = 0.0009). **(B, C)**With the increasing level of M-CSF, there was no significant difference in the invasive ability of PC3 cells between group 0ng/ml and 1.0ng/ml, but the invasion ability of 1.5ng/ml group was significantly enhanced(P < 0.001). **(D, E)**With the increase level of M-CSF, there was no significant difference in the migration ability of PC3 cells between group 0ng/ml and 1.0ng/ml during the whole 36h, but the migration ability of 1.5ng/ml group was significantly enhanced(P < 0.001) since 24h and 36h.

In order to further clarify the effect of M-CSF on PCa, we intervened prostate cancer PC3 cell line by setting M-CSF concentration gradient (0ng/ml, 1.0ng/ml, 1.5ng/ml) for 48 hours, and further observed changes by CCK8, invasion and wound healing assay. After treated with M-CSF concentration gradients of 0ng/l, 1.0ng/l and 1.5ng/ml for 48 hours, the cell viability of PC3 cells was significantly increased with the increase of M-CSF concentration (Fig 1A).

In the Transwell invasion assay (Fig 1B), we observed that with the increase of M-CSF concentration, there was no significant difference in the invasive ability of PC3 between 0ng/ml and 1.0ng/ml, but in the 1.5ng/ml group, the invasive ability of PC3 was significantly enhanced (Fig 1C, P < 0.001).

We further carried out wound healing assay on different groups of PC3 to evaluate the change of cell migration ability (Fig 1D). The results showed that with the increase of M-CSF concentration, the migration ability of PC3 had no significant difference between 0ng/ml and 1.0ng/ml, but in 1.5ng/ml group, the migration ability of PC3 was significantly enhanced (Fig 1E, P < 0.001).

### Proteomic screening for prostate cancer-specific downstream targets of M-CSF

PC3 cells from the experimental group (with three replicates: PC3 T1, T2, T3; cultured in 10% 1640 medium supplemented with 1.5 ng/mL M-CSF) and the control group (with three replicates: PC3 NC1, NC2, NC3; cultured normally in 10% 1640 medium) were collected for protein extraction and mass spectrometry analysis. Quality control was performed on the extracted proteins to ensure the reliability and accuracy of subsequent experiments.

As shown in S1A Fig, a total of 54,774 peptides (Identified peptides) were detected. After removing duplicates, 53,963 peptides (Unique peptides) were obtained, and 7,329 proteins (Identified proteins) were ultimately resolved through specific peptides, with 7,298 proteins (Comparable proteins) available for quantitative comparison. Subsequent quality control assessments included: peptide length distribution (S1B Fig), peptide count distribution (S1C Fig), and protein coverage distribution (S1D Fig).

Since we established three experimental groups and three control groups respectively, it was necessary to further evaluate whether the quantitative results from biological/technical replicate samples demonstrated statistical consistency across groups. For this purpose, we employed three statistical methods: Pearson’s Correlation Coefficient (PCC, S2A Fig), Principal Component Analysis (PCA, S2B Fig), and Relative Standard Deviation (RSD, S2C Fig) to assess sample repeatability. The results indicated high consistency among replicate samples within each group.

To investigate the distribution and variability of protein intensity values across different samples, the protein intensity values for each sample were visualized using box plots (S3A Fig), violin plots (S3B Fig), distribution histograms (S3C Fig), and density distribution curves (S3D Fig). The results consistently indicated that the mean values across samples were generally at a similar level, reflecting high quality of the original protein samples.

To explore the functional characteristics of different proteins, comprehensive functional annotation was performed on the identified proteins. The annotation methods included COG/KOG, protein domain, KEGG pathway, GO, Wikipathways, transcription factor (TF), Reactome, and HallMark. The resulting protein annotation outcomes are illustrated in S4 Fig.

After functional annotation, differentially expressed proteins (DEPs) between the experimental and control groups were compared. The final screening results are shown in S5 Fig: a total of 95 DEPs were identified, among which 69 were upregulated and 26 were downregulated.

The 95 screened differential proteins were further classified into functional functions, and the results are shown in S6 Fig: GO secondary classification shows the functions and attributes of the differential proteins (S6A Fig), subcellular localization classification shows the differential proteins in cells. Location (S6B Fig), COG/KOG classification and KEGG pathway enrichment analysis further explain the functions of the differential proteins (S6C and S6D Fig).

After further comprehensive analysis of the differences in the screened differential proteins, the functions involved, and the pathways involved, ATOX1 related to copper death pathway, HSBP1 related to iron death, SUMO3 gene related to ubiquitin modification, and PCLAF/KIAA0101 gene, which promotes tumor progression and participates in ubiquitin modification, were selected as key genes and further verified in subsequent experiments.

### PCLAF was selected as a downstream key protein among specific targets

**Fig 2:**
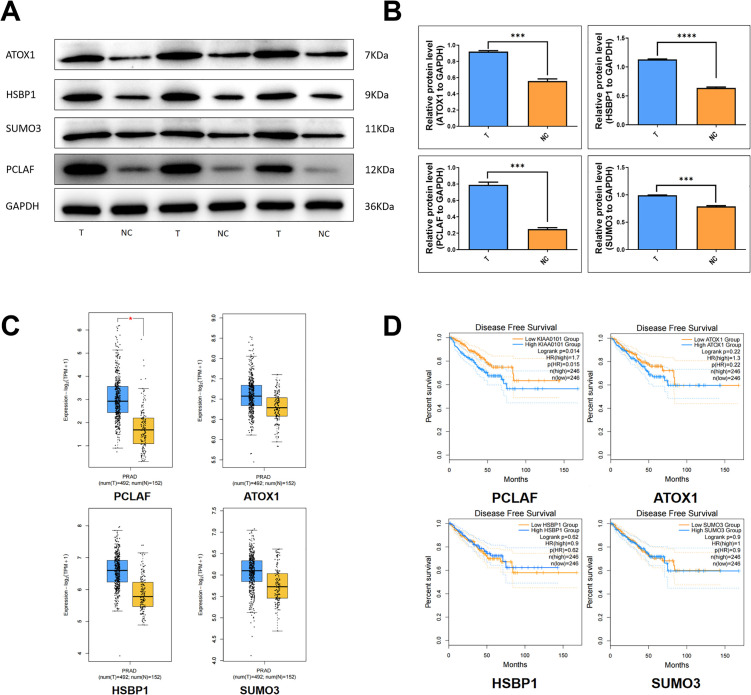
Expression differences of differential proteins KIAA0101, ATOX1, HSBP1, and SUMO3 in tumor vs. normal tissues, their expression in TCGA-PRAD, and predictive value for disease-free survival (DFS). **(A, B)** Among the four differentially expressed proteins, only KIAA0101 showed significantly elevated expression in tumor tissues, while the other three proteins demonstrated no notable changes. **(C)** This expression pattern and differential behavior align with the trends observed in TCGA-PRAD. **(D)** Furthermore, among these four proteins, only KIAA0101 exhibited predictive value for disease-free survival (DFS) in prostate cancer (PCa), with higher expression levels correlating with poorer DFS outcomes.

Among the key genes screened through proteomics, we further conducted expression validation. Western blot analysis was performed on the two cell groups (+M-CSF group and normal culture group) with three biological replicates. The results showed that among the four differential proteins, only PCLAF/KIAA0101 exhibited a statistically significant difference between the two groups (P < 0.001), while ATOX1 (P = 0.087), HSBP1 (P = 0.2098), and SUMO3 (P = 0.8063) showed no significant statistical differences (Fig 2A, 2B). This indicates that with the increase in M-CSF, only the expression level of PCLAF/KIAA0101 was upregulated.

To further validate the expression of the four key genes in prostate cancer and their predictive value for disease-free survival (DFS), GEPIA2 was utilized for verification. The expression differences are shown in Fig 2C: In TCGA-PRAD, KIAA0101 showed a significant expression difference between tumor and non-tumor groups (P < 0.05), while ATOX1, HSBP1, and SUMO3 exhibited no significant expression differences between the two groups (P > 0.05). The DFS differences are shown in Fig 2D: KIAA0101 demonstrated certain predictive value for DFS in prostate cancer, with the high-expression group showing significantly reduced DFS (P = 0.015); whereas no DFS differences were observed between high and low expression groups of ATOX1 (P = 0.22), HSBP1 (P = 0.62), and SUMO3 (P = 0.9).

### Validation of KIAA0101 expression differences in tumor and subgroup classifications using tissue microarray

Tissue microarray was employed to validate the expression of PCLAF/KIAA0101 in tumor tissues. A total of 79 patient tissue samples were included, comprising 9 cases of benign prostatic hyperplasia (BPH) and normal prostate tissues, along with 69 cases of localized prostate cancer (n = 0, m = 0). The Gleason grade groups were distributed as follows: 7 cases in group 1, 8 in group 2, 7 in group 3, 30 in group 4, and 17 in group 5. One case was excluded due to undetermined malignancy. According to the International Society of Urological Pathology (ISUP) grading system, the prostate cancer cases were classified as: 7 low-risk, 15 intermediate-risk, and 47 high-risk cases.

**Fig 3:**
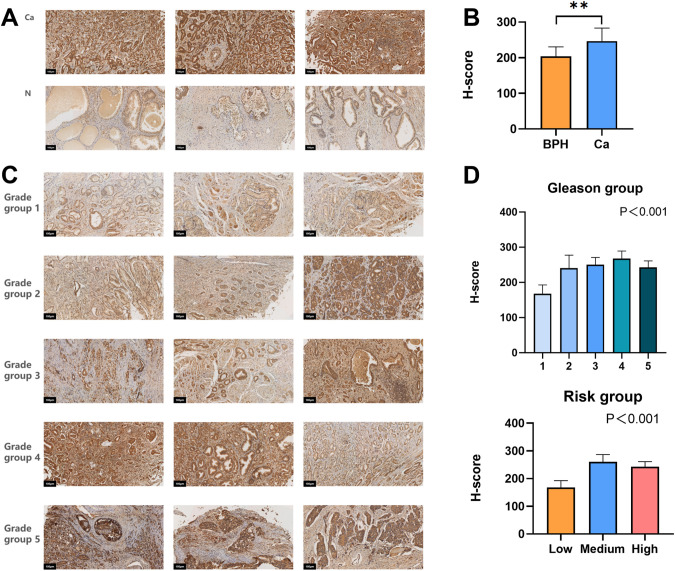
Differential expression of PCLAF/KIAA0101 in benign vs malignant tissues, and across gleason grade groups and ISUP risk groups. **(A)**Representative immunohistochemical (IHC) staining of PCLAF/KIAA0101 in benign prostate tissues and localized prostate cancer tissues. **(B)**Quantitative analysis of PCLAF/KIAA0101 expression based on H-SCORE evaluation. PCLAF/KIAA0101 expression was significantly elevated in tumor tissues compared to non-tumor tissues (**P = 0.0013). **(C)**Differential expression of PCLAF/KIAA0101 across prostate cancer Grade Groups (Gleason Score-based classification). Statistical significance was observed among different groups (***P < 0.001). **(D)**Differential expression of PCLAF/KIAA0101 across ISUP risk classifications. Expression levels showed statistically significant variation among risk groups (***P < 0.001).

Analysis of H-SCORE results from immunohistochemical staining of PCLAF/KIAA0101 in 9 non-tumor patients and 69 patients with localized prostate cancer is shown in Fig 3A. The results demonstrate significantly elevated PCLAF/KIAA0101 expression in tumor tissues (P = 0.0013, Fig 3B). The differential expression of PCLAF/KIAA0101 across Grade groups and ISUP risk groups is shown in Fig 3C, 3D: statistically significant differences in KIAA0101 expression levels were observed among different groups in both Grade group and ISUP classifications (P < 0.001). These findings indicate that PCLAF/KIAA0101 is highly expressed in tumor tissues and increases with tumor progression.

### Nude mouse xenograft experiment investigating the promoting effect of PCLAF/KIAA0101 upregulation on prostate cancer cells

**Fig 4:**
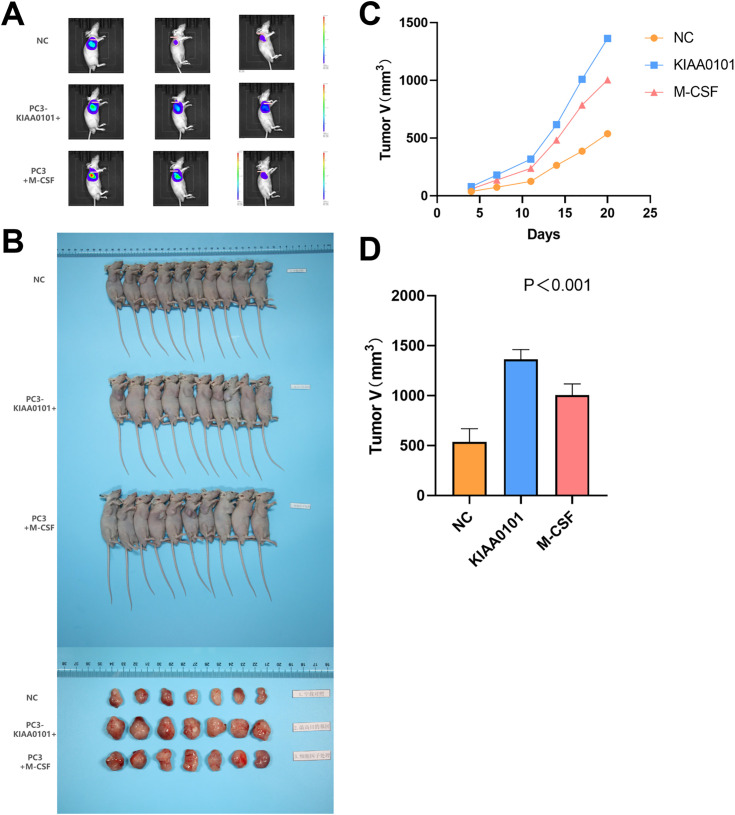
Promoting effects of KIAA0101 overexpression and M-CSF on tumor growth in PC3 cells in vivo. **(A)**In vivo bioluminescence imaging of subcutaneous xenograft tumors in nude mice. Tumor models were established using PC3 empty vector control cells, PC3-KIAA0101 + overexpression cells, and PC3 cells with M-CSF treatment. Imaging was performed one week after tumor inoculation. **(B)**Representative images of xenograft tumors at the experimental endpoint. From left to right: tumors from PC3 empty vector control group, PC3-KIAA0101 + overexpression group, and PC3 + M-CSF treatment group. **(C)**Tumor growth curves of the three experimental groups. Both PC3-KIAA0101+ and PC3 + M-CSF groups showed significantly enhanced tumor growth compared to the empty vector control group (***P < 0.001). **(D)**Final tumor weights of xenograft tumors from the three experimental groups. Statistical analysis revealed significant increases in tumor weight in both PC3-KIAA0101+ and PC3 + M-CSF groups compared to the control group (***P < 0.001).

Based on the results from cell experiments and immunohistochemical analysis, we further validated the cancer-promoting effect of KIAA0101 using a xenograft tumor model in nude mice. Subcutaneous tumor models were established in the axillary region of BALB/c nude mice using three groups: the PC3 control group, the PC3-KIAA0101 + group, and the PC3 + M-CSF group. The specific groupings were as follows: 1) The PC3 empty vector group, consisting of normally cultured cell lines serving as the control, received saline injections every three days; 2) The PC3-KIAA0101 + group, established via lentiviral transduction to overexpress KIAA0101, also received saline injections every three days; 3) The PC3 + M-CSF group was initially inoculated with normally cultured PC3 cells, followed by local injections of M-CSF at a dose of 1 mg/kg every three days. In vivo imaging results one week after tumor inoculation are shown in Fig 4A.

The results of the tumor xenograft experiment at the endpoint are presented in Fig 4B–4D. In the PC3 xenograft model, local injection of M-CSF significantly increased tumor growth volume, showing a statistically significant difference compared to the empty vector control group (P < 0.001). The PC3-KIAA0101 + group exhibited an even more pronounced increase in tumor volume, with a statistically significant difference compared to the control group (P < 0.001). These findings preliminarily indicate that both M-CSF supplementation and KIAA0101 overexpression significantly enhance the proliferative and invasive capabilities of PC3 cells. Furthermore, the results demonstrate that M-CSF supplementation and KIAA0101 overexpression produce analogous biological effects in PC3 cells.

**Fig 5:**
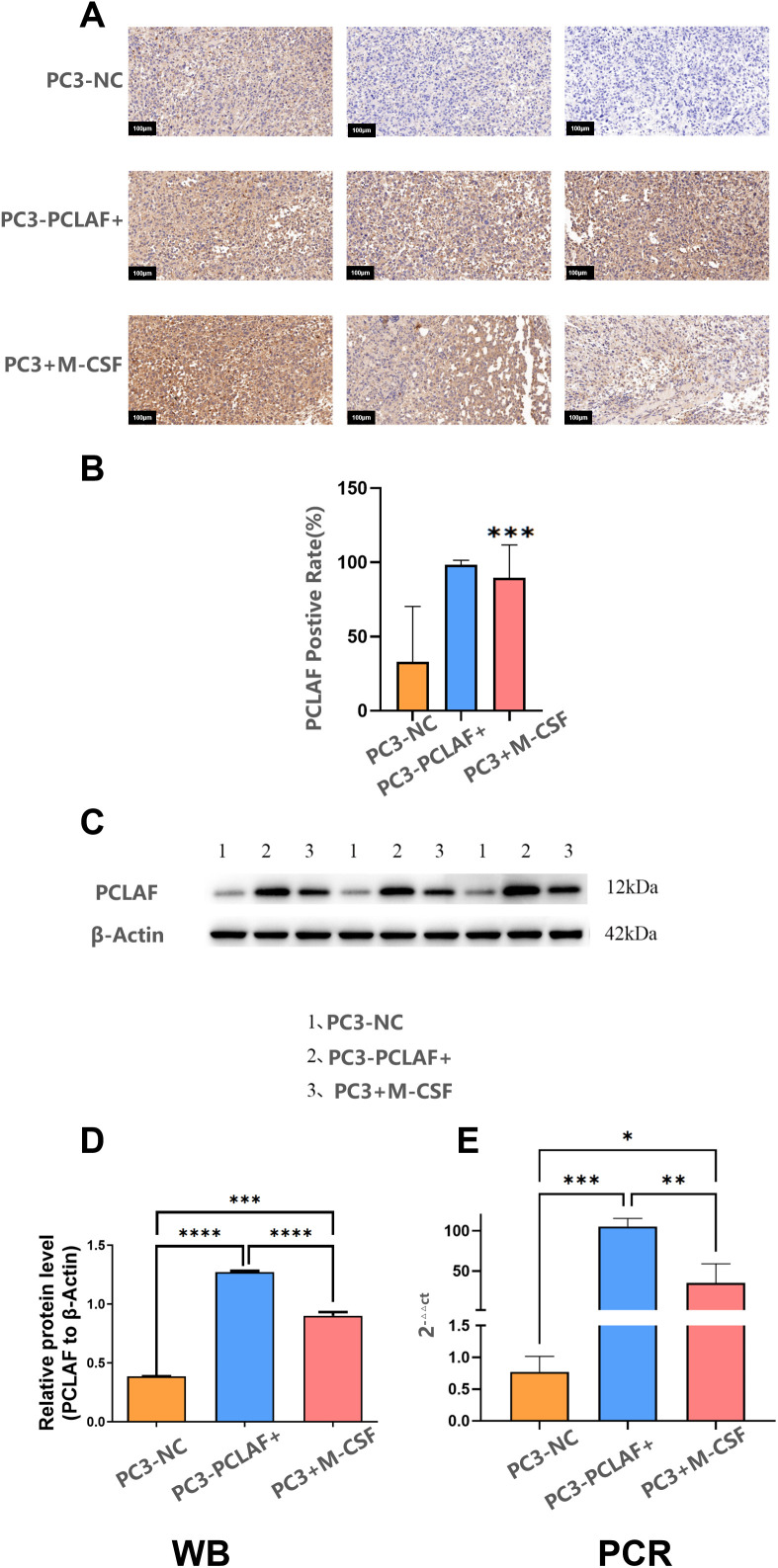
Expression of KIAA0101 in xenograft tumors from different experimental groups (IHC, western blot, PCR). **(A)**Representative immunohistochemical (IHC) staining of KIAA0101 in xenograft tumor tissues from the three experimental groups (Scale bar: 50 μm). **(B)**Quantitative analysis of KIAA0101 protein expression based on IHC staining intensity. KIAA0101 expression showed the pattern: PC3-KIAA0101+ > PC3 + M-CSF > empty vector control (*P < 0.05). **(C)**Western blot analysis of KIAA0101 protein expression in xenograft tumors from the three experimental groups. **(D)**Quantitative analysis of KIAA0101 protein levels from Western blot results. Densitometric analysis confirmed the expression pattern: PC3-KIAA0101+ > PC3 + M-CSF > empty vector control (*P < 0.05). **(E)**Quantitative PCR analysis of KIAA0101 mRNA expression in xenograft tumors. Gene expression levels followed the pattern: PC3-KIAA0101+ > PC3 + M-CSF > empty vector control (*P < 0.05).

Further IHC, Western blot (WB), and PCR analyses were performed on the xenograft tumors to determine KIAA0101 expression levels across the experimental groups, as shown in Fig 5: (1)IHC results are presented in Fig 5A, 5B. (2)WB results are shown in Fig 5C, 5D. (3)PCR results are displayed in Fig 5E.

All results consistently demonstrated the following pattern of PCLAF/KIAA0101 expression: PC3-KIAA0101 + group > PC3 + M-CSF group > empty vector control group (P < 0.05). These findings indicate that upregulation of M-CSF in the xenograft model leads to increased PCLAF/KIAA0101 expression. Furthermore, PCLAF/KIAA0101 expression levels showed a positive correlation with tumor growth rate and volume, suggesting that M2 macrophage-mediated promotion of prostate cancer may be achieved through M-CSF-induced upregulation of PCLAF/KIAA0101.

## Discussion

Prostate cancer has become the most common malignancy among men in multiple countries, and its mortality rate is rising annually. Immune system dysregulation has been identified as a major cause of tumorigenesis in various cancer types. The TME of prostate cancer contains a complex network of immunosuppressive molecules, and this immunosuppressive TME renders patients with advanced disease, such as castration-resistant prostate cancer (CRPC), poorly responsive to immunotherapy [18]. Therefore, in-depth research into how the immune system promotes the initiation and progression of prostate cancer, and the identification of new therapeutic targets, is particularly important.

Colony-stimulating factors are crucial cytokines with diverse functions in the body. Beyond promoting the proliferation and differentiation of hematopoietic stem cells, they also act on various mature cells. Their role in tumor initiation, development, detection, and treatment has been a hot topic in cancer immunology research in recent years. Campbell MJ et al. [19] found that highly proliferative macrophages and their highly secreted cytokines, such as M-CSF, in breast cancer are associated with high-grade, malignant subtypes and poor clinical prognosis, suggesting that M2 macrophages and M-CSF are key factors promoting tumor progression. Klemm F et al. [20], through abundance studies on tissue microarrays from small cell lung cancer, non-small cell lung cancer, melanoma, colorectal cancer, breast cancer, and renal cell carcinoma, found that the M-CSF receptor tends to be highly expressed across all these cancer types. They validated that most M-CSF receptors originate from the monocyte/macrophage lineage and further demonstrated, using mouse models, that M-CSF receptor expression is elevated in brain metastases. This suggests that M-CSF and its receptor play a significant cancer-promoting role in malignant tumors, especially those with high malignancy and metastatic potential. Zhang Z et al. [21] demonstrated that high M-CSF expression is detectable in 76% of primary ovarian cancers and 70% of ovarian cancer metastases, indicating a positive correlation between M-CSF expression levels and the invasive and metastatic capacity of ovarian cancer. Furthermore, both the M-CSF receptor and ligand are involved in trophoblast cell invasion. The M-CSF receptor is detectable in 90% of primary lesions and 80% of metastatic lesions, and co-expression of the M-CSF ligand and receptor can serve as an independent risk factor for metastatic ovarian cancer, shortening the average time to tumor recurrence by 11 months. In this study, we found that M2 macrophages co-cultured with prostate cancer cells secreted significantly higher levels of M-CSF. We further elucidated that the specific mechanism involves the upregulation of the MYC pathway via MS4A6A. Furthermore, by performing proteomic analysis on prostate cancer cells treated with or without M-CSF, we identified 95 proteins with significantly altered expression after M-CSF treatment. This indicates that prostate cancer cells can promote M2 macrophages to secrete M-CSF, and M-CSF, in turn, promotes a series of functional protein changes in prostate cancer cells, further driving tumor progression.

Omics analysis encompasses a series of biological and bioinformatics methods primarily used for the systematic study of the structural relationships and dynamic functions of organisms and their components, including genomics, proteomics, metabolomics, transcriptomics, and more. Since gene function is ultimately executed by proteins, and protein expression involves complex regulatory processes such as transcription and translation, the levels of gene transcription and translation cannot fully represent protein expression, especially for low-abundance proteins, protein modifications, and subcellular localization. Proteomics can often provide better explanations for disease initiation and progression compared to genomics. Consequently, proteomics has been increasingly applied in recent years for precise research into tumor mechanisms. The team of Petralia F [22] conducted a multi-omics study on the tumor immune microenvironment using 1056 pan-cancer samples. Through proteomics and phosphoproteomics, they further elucidated mechanisms of immune infiltration and tumor immune escape, finding that the functional state of infiltrating immune cells and tumor-specific activation modes are crucial for resistance to cancer immunotherapy. Vasaikar S et al. [23] performed the first large-cohort proteomic study of colon cancer in 2019. While discovering new tumor markers and targets, they also explained that Rb protein phosphorylation is associated with colon cancer proliferation and extended cell cycle, identifying Rb phosphorylation as a new potential targeting site for colorectal cancer. The application of proteomics in prostate cancer research has also garnered increasing attention. Sinha A et al. [24] found that in 76 patients with sporadic localized prostate cancer, 8 proteins were associated with tumor size and 7 proteins were linked to tumor aggressiveness (specifically, the presence of intraductal carcinoma pathology). They further discussed that in prognostic models using biochemical recurrence as the endpoint, protein abundance hazard ratios had a wider dynamic range than mRNA abundance hazard ratios. This suggests that in studying localized prostate cancer prognosis, protein translation dysregulation, modification, and circuit regulation collectively contribute to aggressive progression, and biomarkers screened by proteomics hold greater clinical value. Latonen L et al. [25], through proteomic studies of patients with benign prostatic hyperplasia, primary prostate cancer, and CRPC, revealed that in prostate cancer, miRNA targets lack correlation at the transcript level but show strong correlation at the translation level. This indicates that the stability of genomic and transcriptomic variations is an important factor in prostate cancer progression. Cytokines are a crucial pathway through which macrophages exert effects on tumor cells, and the modes of action are diverse and complex. Therefore, in this study, we treated two groups of prostate cancer PC3 cells with M-CSF and then performed proteomic analysis on these cells to screen for targets that undergo significant changes upon M-CSF action. In the protein quality control results, we observed that compared to tissue samples used in omics analyses, cell samples yielded better quality control results. Furthermore, there were no extensive differences in identifiable peptides and proteins between the two groups. This indicates that in subsequent screening analyses, expression differences of key genes between the two cell groups are more likely attributable to M-CSF, significantly enhancing the reliability of the screening results. By further filtering based on the fold change of differentially expressed proteins, we selected the PCLAF protein associated with tumor proliferation, the ATOX1 protein associated with cuproptosis, the HSBP-1 protein associated with ferroptosis, and the SUMO-3 protein associated with ubiquitination. In subsequent survival analysis and expression validation, the ATOX1, HSBP-1, and SUMO3 genes did not demonstrate prognostic value or consistent expression differences. In contrast, high expression of the PCLAF gene was not only associated with worse prostate cancer prognosis but also showed significant expression differences in in vitro experiments, consistent with the proteomics results. Therefore, the PCLAF gene was ultimately identified as the key protein for further analysis.

The PCLAF gene typically functions in regulating the cell cycle in normal tissues and also plays important biological roles such as DNA synthesis and repair. In recent years, numerous studies have found that PCLAF acts as an oncogene in various cancers, and its elevated expression is positively correlated with adverse prognostic events like tumor metastasis, distant metastasis, and shortened survival. Tantiwetrueangdet A et al. [17] found that in liver cancer, the PCLAF protein tends to be overexpressed, and its expression significantly correlates with both P53 and Ki-67. In monitoring the prognosis of hepatocellular carcinoma, combined detection of PCLAF, P53, and Ki-67 could serve as a potential biomarker and basis for developing new HCC treatments. Shaw GL et al. [26], studying 27 patients who received rapid androgen deprivation therapy with Degarelix prior to radical prostatectomy, found that PCLAF expression decreased early during treatment. They also noted that this gene has binding sites for the androgen receptor (AR), and the upregulation of PCLAF, FAM129A, and RAB27A might promote AR expression, potentially representing a mechanism for prostate cancer cell survival under castrate conditions. In this study, we found that the PCLAF protein in prostate cancer cells was significantly upregulated with increasing M-CSF concentration and was associated with poor prognosis in prostate cancer patients. Through in vitro and in vivo experiments, we further validated that upregulation of PCLAF significantly increased tumor growth rate. This suggests that within the prostate cancer microenvironment, M2 macrophages may promote prostate cancer progression by upregulating M-CSF, which in turn enhances PCLAF expression. While reviewing relevant literature, we found that PCLAF is closely related to PCNA, potentially involving ubiquitination in prostate cancer. In subsequent research, we will delve deeper into this direction to uncover the underlying mechanisms.

In the previous two parts of our research, we discovered and validated that within the prostate cancer immune microenvironment, M2 macrophages promote M-CSF secretion via the upregulation of MS4A6A-MYC. In this part, we provide evidence that the tumor-promoting effect of M-CSF on prostate cancer may be mediated through the upregulation of the PCLAF gene. Our in vitro and in vivo experiments support this hypothesis, although definitive proof would require genetic loss-of-function models such as PCLAF knockout. We acknowledge this as a limitation of the current study and plan to address it in future work. In future studies, we will further investigate the specific signaling pathways through which PCLAF acts in prostate cancer. While attempting to elucidate the mechanisms of the prostate cancer immune microenvironment, we also aim to explore new potential targets and provide new insights for immunotherapy.

## Conclusions

M-CSF induced a series of protein changes in prostate cancer PC3 cells, with PCLAF expression being upregulated upon M-CSF stimulation. PCLAF expression is significantly elevated in tumor tissues, positively correlated with tumor malignancy, and associated with poorer survival prognosis. In vivo experiments indicated that PCLAF expression levels are positively correlated with tumor growth rate and volume, suggesting that M-CSF promotes prostate cancer progression potentially through upregulating PCLAF expression. However, causal evidence awaits future studies using PCLAF knockout models.

## Supporting information

S1 Table Grouping and treatment protocols in the xenograft experiment. (DOCX)

S1 Fig Proteins identified by mass spectrometry analysis in M-CSF-treated PC3 cells and quality control results. (A)The mass spectrometry analysis identified a total of 54,774 peptide sequences. After removing duplicate detections, 53,963 unique peptide sequences were obtained, resulting in the identification of 7,329 proteins and 7,298 quantitatively comparable proteins. (B) The peptide lengths were predominantly distributed between 7–20 amino acids, consistent with the typical length range for enzymatic digestion and mass spectrometry fragmentation. This indicates that the identified peptides met quality control standards in terms of length distribution. (C) The distribution of peptide counts showed that most proteins corresponded to two or more peptides. In quantitative analysis, having multiple specific peptides per protein enhances the reliability and accuracy of the quantification results. (D) The coverage of most detected proteins was ≤ 30%.
(DOCX)

S2 Fig Replicate assessment of proteins identified by mass spectrometry in M-CSF-treated PC3 cells. (DOCX)

S3 Fig Distribution of sample protein intensity values. (DOCX)

S4 Fig Protein function annotation results from different identification methods. (DOCX)

S5 Fig Differential gene screening results between M-CSF treated group and normal culture group. (DOCX)

S6 Fig Differential protein function, localization and pathway analysis results. (DOCX)

S1 File Raw_images. Raw blot and gel images of WB. (PDF)

S2 File Data for WB Raw data of WB. (RAR)
