# Supplementary material for: Tumor-associated M2 macrophages promote prostate cancer invasion through the M-CSF-PCLAF pathway
Source: PLoS One. 2026 Jun 22;21(6):e0351858. doi: 10.1371/journal.pone.0351858 (PMC13286207; doi:10.1371/journal.pone.0351858)
Supplement: S1 Table — (DOCX) [file pone.0351858.s001.docx]

**Supplementary Table.1 Grouping and Treatment Protocols in the Xenograft Experiment**

| Groups | Animal Number | Cell Viability | Inoculation Density | Inoculation Volume | Total Cell Suspension Volume | Ratio with Matrigel |
| --- | --- | --- | --- | --- | --- | --- |
| NC | 10 | ＞90% | 8×10^7^/ml | 100μL | 1.2mL | 1:1 mixed with Matrigel for inoculation |
| PC3-KIAA0101+ | 10 | ＞90% | 8×10^7^/ml | 100μL | 1.2mL | 1:1 mixed with Matrigel for inoculation |
| PC3+M-CSF | 10 | ＞90% | 8×10^7^/ml | 100μL | 1.2mL | 1:1 mixed with Matrigel for inoculation |
