# Supplementary material for: Tumor-associated M2 macrophages promote prostate cancer invasion through the M-CSF-PCLAF pathway
Source: PLoS One. 2026 Jun 22;21(6):e0351858. doi: 10.1371/journal.pone.0351858 (PMC13286207; doi:10.1371/journal.pone.0351858)
Supplement: S1 Fig — (A)The mass spectrometry analysis identified a total of 54,774 peptide sequences. After removing duplicate detections, 53,963 unique peptide sequences were obtained, resulting in the identification of 7,329 proteins and 7,298 quantitatively comparable proteins. (B) The peptide lengths were predominantly distributed between 7–20 amino acids, consistent with the typical length range for enzymatic digestion and mass spectrometry fragmentation. This indicates that the identified peptides met quality control standards in terms of length distribution. (C) The distribution of peptide counts showed that most proteins corresponded to two or more peptides. In quantitative analysis, having multiple specific peptides per protein enhances the reliability and accuracy of the quantification results. (D) The coverage of most detected proteins was ≤ 30%. (DOCX) [file pone.0351858.s002.docx]

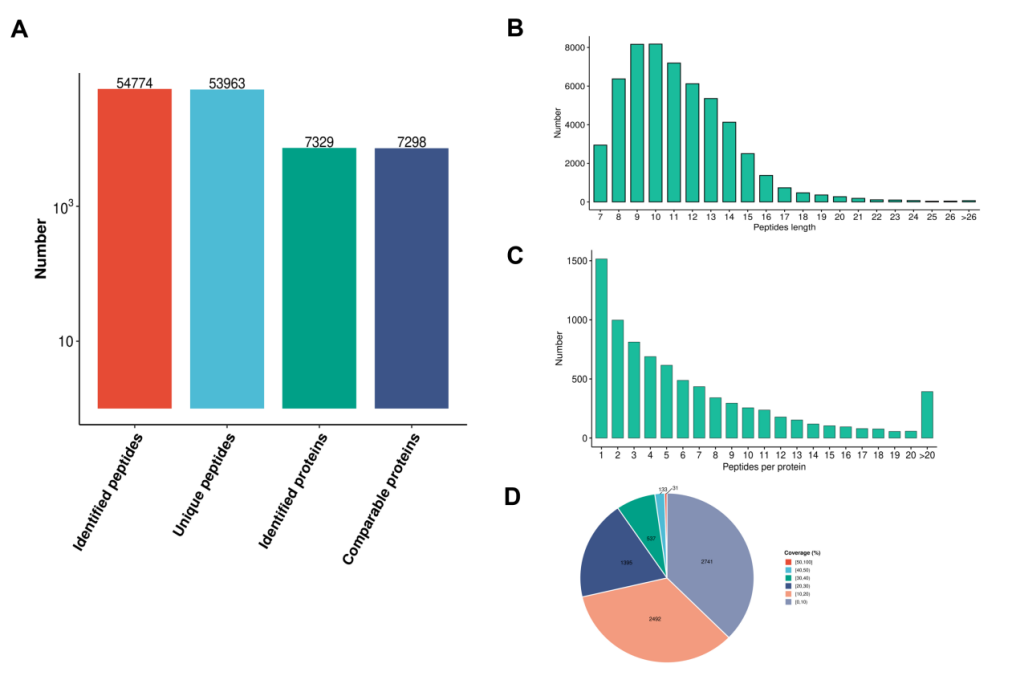


**Supplementary figures.1 Proteins Identified by Mass Spectrometry Analysis in M-CSF-Treated PC3 Cells and Quality Control Results.** (A)The mass spectrometry analysis identified a total of 54,774 peptide sequences. After removing duplicate detections, 53,963 unique peptide sequences were obtained, resulting in the identification of 7,329 proteins and 7,298 quantitatively comparable proteins. (B) The peptide lengths were predominantly distributed between 7–20 amino acids, consistent with the typical length range for enzymatic digestion and mass spectrometry fragmentation. This indicates that the identified peptides met quality control standards in terms of length distribution. (C) The distribution of peptide counts showed that most proteins corresponded to two or more peptides. In quantitative analysis, having multiple specific peptides per protein enhances the reliability and accuracy of the quantification results. (D) The coverage of most detected proteins was ≤30%.
