# Supplementary material for: Tumor-associated M2 macrophages promote prostate cancer invasion through the M-CSF-PCLAF pathway
Source: PLoS One. 2026 Jun 22;21(6):e0351858. doi: 10.1371/journal.pone.0351858 (PMC13286207; doi:10.1371/journal.pone.0351858)
Supplement: S6 Fig — (DOCX) [file pone.0351858.s007.docx]

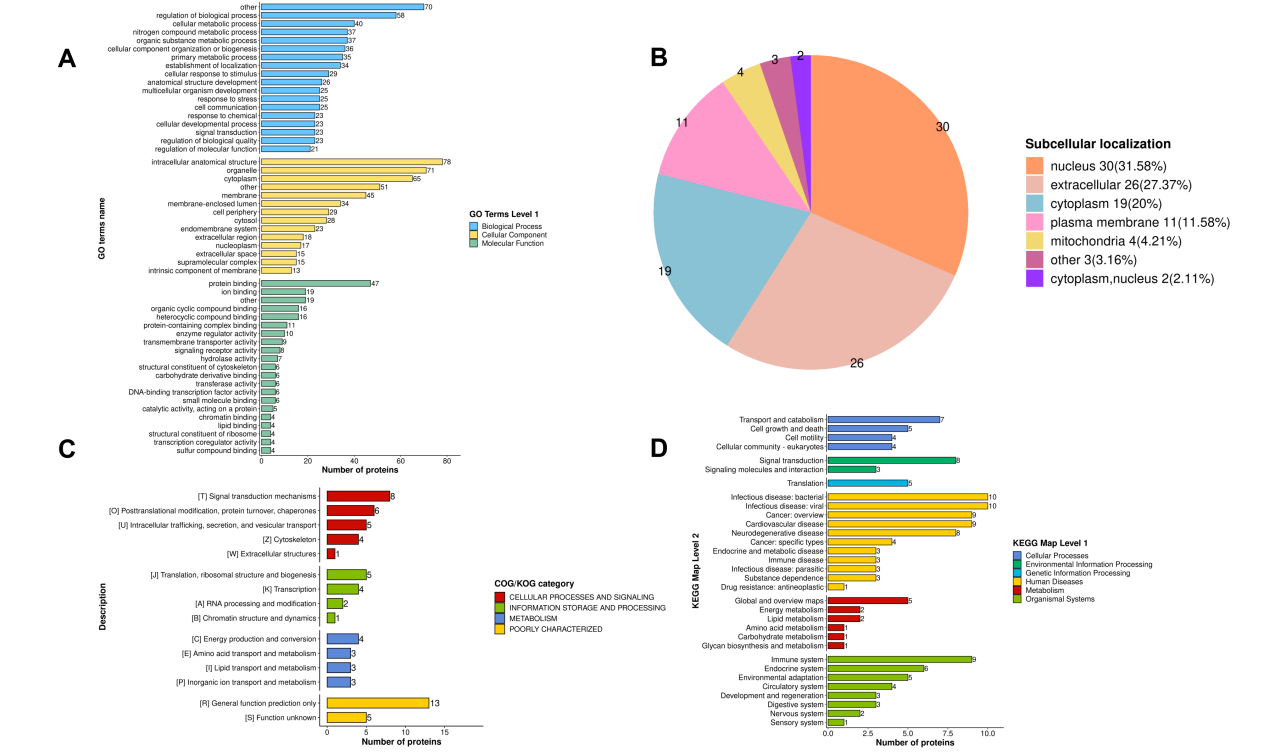


**Supplementary figures.6 Differential protein function, localization and pathway analysis results**
