# Supplementary material for: Tumor-associated M2 macrophages promote prostate cancer invasion through the M-CSF-PCLAF pathway
Source: PLoS One. 2026 Jun 22;21(6):e0351858. doi: 10.1371/journal.pone.0351858 (PMC13286207; doi:10.1371/journal.pone.0351858)

Raw WB images for Figures.2

ATOX1

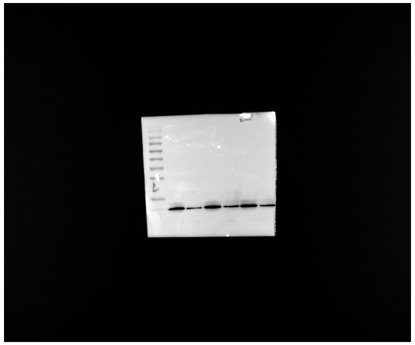

GAPDH

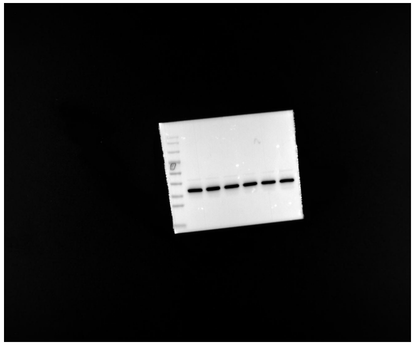

HSBP1

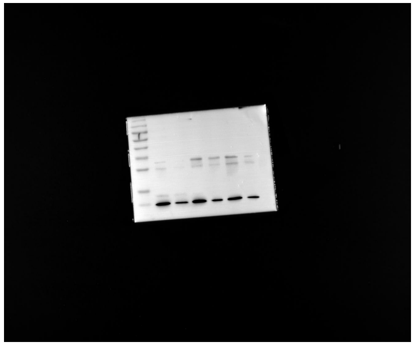

PCLAF

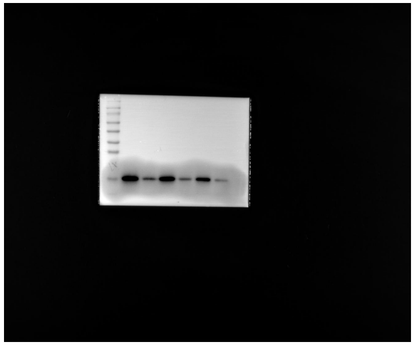

SOMO3

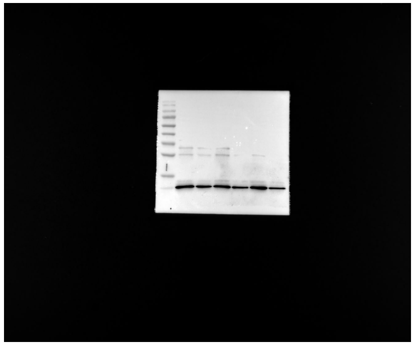

Raw WB images for Figures.5

PCLAF(KIAA0101)

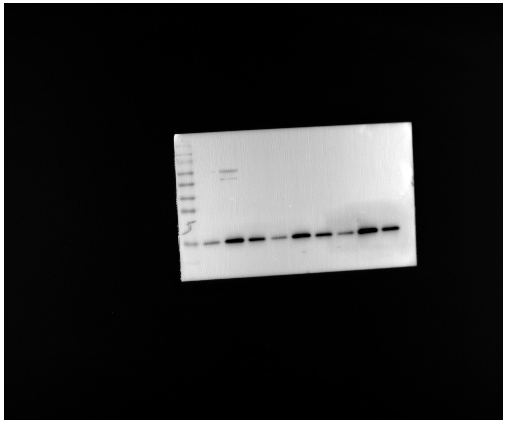

β-Actin

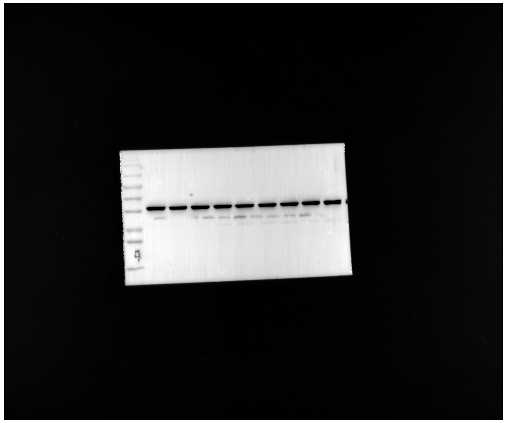

Supplement: S1 File — (PDF) [file pone.0351858.s008.pdf]
